# Sarcopenia is associated with cardiovascular risk in men with COPD, independent of adiposity

**DOI:** 10.1186/s12931-022-02109-3

**Published:** 2022-07-13

**Authors:** Ah Young Leem, Young Sam Kim, Kung Soo Chung, Moo Suk Park, Young Ae Kang, Young-Mok Park, Ji Ye Jung

**Affiliations:** Division of Pulmonology and Critical Care Medicine, Department of Internal Medicine, Severance Hospital, Yonsei University College of Medicine, 50-1 Yonsei-ro, Seodaemun-gu, Seoul, 03722 Republic of Korea

**Keywords:** Chronic obstructive pulmonary disease, Sarcopenia, Cardiovascular disease, Risk, Adiposity

## Abstract

**Background:**

Sarcopenia is a well-established risk factor for atherosclerotic cardiovascular disease (ASCVD), but its relationship with chronic obstructive pulmonary disease (COPD) has not been fully determined. This study is aimed to investigate the association between sarcopenia and risk for ASCVD in patients with COPD, independent of central obesity and fat mass.

**Methods:**

Data regarding 704 men with COPD (mean age: 63.4 years) were extracted from the 2008 to 2011 Korean National Health and Nutrition Examination Surveys. Sarcopenia index and fat mass were assessed using dual-energy X-ray absorptiometry. Sarcopenia was defined according to the presence of sarcopenia index values < 1 standard deviation from the cutoff (0.774) among the study participants. ASCVD risk was evaluated using American College of Cardiology/American Heart Association guidelines. High probability of ASCVD was defined as ASCVD risk > 20%.

**Results:**

The quartile-stratified sarcopenia index was negatively associated with ASCVD risk (*P* < 0.001). ASCVD risk and prevalence of high ASCVD risk were significantly greater in sarcopenic participants than in non-sarcopenic participants, regardless of central obesity and fat mass (all *P* < 0.001). Multivariate regression analyses demonstrated an independent association between sarcopenia and ASCVD risk (estimated ± standard error = 3.63 ± 0.77%, *P* < 0.001) and high ASCVD risk (odds ratio [OR] = 2.32, 95% confidence interval [CI] 1.05–5.15, *P* = 0.039). Furthermore, sarcopenia was an independent factor for high ASCVD risk in participants with moderate to very severe airflow limitation (OR = 2.97, 95% CI 1.06–8.36, *P* < 0.001).

**Conclusions:**

Sarcopenia was significantly associated with an increased risk for ASCVD in men with COPD, independent of central obesity and fat mass. High ASCVD risk was significantly associated with sarcopenia, particularly in participants with moderate to very severe airflow limitation.

**Supplementary Information:**

The online version contains supplementary material available at 10.1186/s12931-022-02109-3.

## Background

The burden of chronic obstructive pulmonary disease (COPD) increases with extrapulmonary impairment. Cardiovascular, musculoskeletal, and psychological conditions are important comorbidities that contribute to symptoms, exacerbations, hospital admissions, and mortality involving patients with COPD [1]. Sarcopenia is a syndrome characterized by the progressive loss of skeletal muscle mass and strength leading to physical disability, poor quality of life, and death [2]. The pathophysiology of sarcopenia in patients with COPD is multifactorial, including disuse atrophy secondary to reduced activity, systemic corticosteroid therapy, cachexia syndrome secondary to inflammation, hormone imbalance, hypoxia, and oxidative stress [1].

Sarcopenia is more prevalent in patients with COPD than in the general population [3]. Moreover, the prevalence of sarcopenia increases with increasing severity of airflow limitation [3]. Sarcopenia is closely associated with various health conditions including osteopenia/osteoporosis, nonalcoholic fatty liver disease, and metabolic syndrome in patients with COPD [3–5]. In the general population, sarcopenia is a well-established risk factor for cardiovascular disease. Individuals with sarcopenic obesity [co-presence of sarcopenia and obesity (defined as a BMI ≥ 25.0 kg/m^2^)] exhibit a greater risk for cardiovascular disease, compared to individuals who have a normal body mass index (BMI) and muscle mass. However, obese but non-sarcopenic individuals do not exhibit an increased risk for cardiovascular disease [6]. By contrast, sarcopenia is associated with cardiovascular disease mortality in older men, although it is not significantly associated with cardiovascular disease events [7]. Muscle strength rather than muscle mass might be more important to predictive of CVD events.

Cardiovascular diseases, particularly ischemic heart diseases, are the leading causes of death in patients with COPD who have mild to moderate airflow limitation. Although there is increasing evidence that sarcopenia is a risk factor for cardiovascular disease, the association between sarcopenia and risk for cardiovascular disease has not been fully determined in patients with COPD. This study investigated the relationship between sarcopenia and risk for cardiovascular disease in patients with COPD, independent of central obesity and fat mass, using data from the Korea National Health and Nutrition Examination Survey (KNHANES).

## Methods

### Study population

This cross-sectional study extracted data from the 2008–2011 KNHANES. The KNHANES is a nationwide, population-based, cross-sectional health examination and survey that is conducted annually by the Division of Chronic Disease Control and Prevention in the Ministry of Health and Welfare to monitor the general health and nutritional characteristics of the general civilian population in South Korea [8]. Participants are randomly selected from 600 randomly selected districts in cities and provinces in South Korea to represent a sample of the Korean population.

**Fig. 1:**
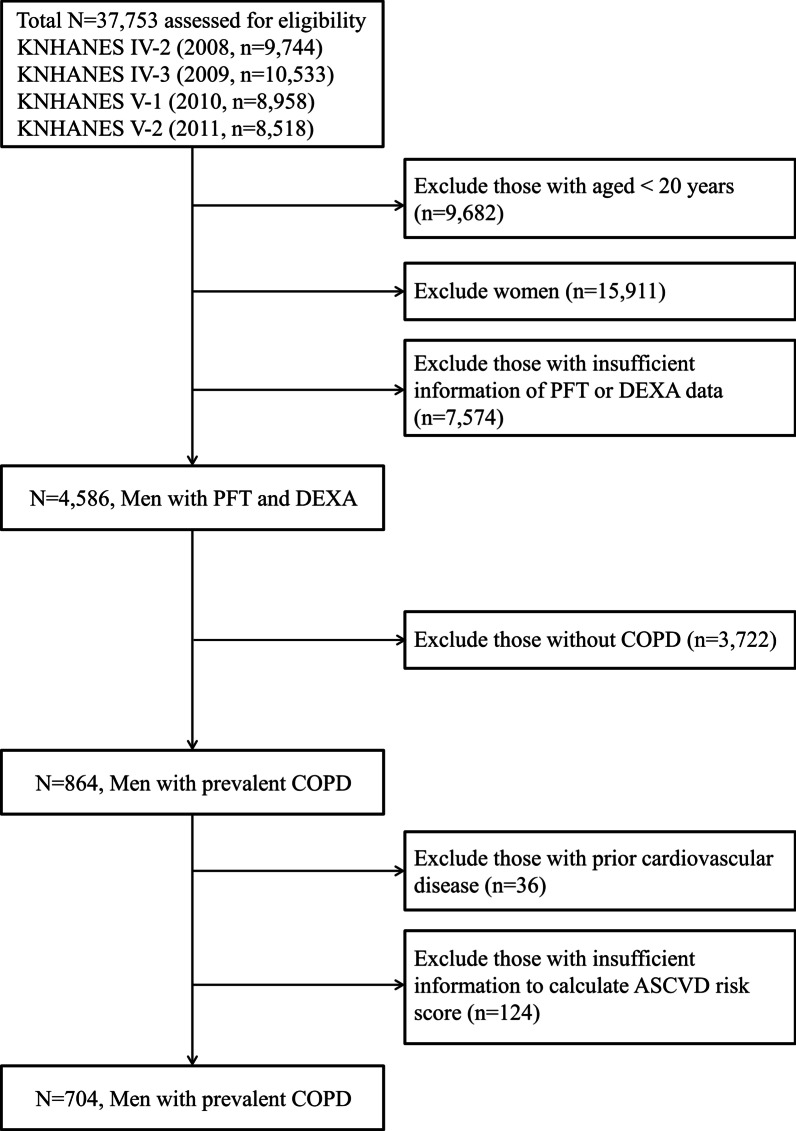
Flow diagram of participant inclusion and exclusion in the KNHANES IV and V. Of the total participants (n = 37,753), 704 men with COPD were ultimately included. *KNHANES* Korea National Health and Nutrition Examination Survey, *ASCVD* atherosclerotic cardiovascular disease, *COPD* chronic obstructive pulmonary disease, *PFT* pulmonary function test, *DEXA* dual-energy X-ray absorptiometry

As described in Fig. 1, of the 37,753 participants in the 2008–2011 KNHANES, we initially selected 864 men with COPD based on spirometry results (ratio of forced expiratory volume in 1 s [FEV_1_]/forced vital capacity [FVC] < 0.7). Of the 864 participants, 160 were excluded because of prior cardiovascular disease history (n = 36) or insufficient information to calculate atherosclerotic cardiovascular disease (ASCVD) risk score (n = 124). Finally, 704 eligible men with COPD were included in this study (Fig. 1). KNHANES data include a medical history, nutritional status, and laboratory tests. The participants’ medical histories were evaluated, including smoking, exercise level, and disease diagnosis or treatment, based on direct interviews and self-reporting. Smoking status was self-reported as non-smoker or current smoker. Regular exercise was defined as > 20 min per session, at least 3 times per week.

### Lung function

Pulmonary function parameters, including FEV_1_, FVC, and the ratio of FEV_1_ to FVC, were assessed using a dry rolling-seal spirometer (model 2130; SensorMedics), in accordance with the standardization criteria of the American Thoracic Society and the European Respiratory Society [9]. COPD was defined as a pre-bronchodilator FEV_1_/FVC ratio < 0.7. Participants with COPD were categorized into two groups according to the degree of airflow limitation. The mild limitation group included participants with FEV_1_ ≥ 80% of predicted value, and the moderate to very severe limitation group included participants with FEV_1_ < 80% of predicted value.

### Measurement of body composition and definitions of sarcopenia and adiposity

Appendicular skeletal muscle (ASM) and fat mass were measured using dual-energy X-ray absorptiometry (QDR 4800A; Hologic Inc., Bedford, MA, USA). The sarcopenia index was calculated as follows: sarcopenia index = total ASM mass (kg)/BMI (kg/m^2^) [5]. Sarcopenia was defined according to the presence of sarcopenia index values < 1 standard deviation (SD) from the mean value of the study participants, according to the previous studies [3, 10]. The cutoff value was 0.774 in this study. Fat mass index (FMI) was calculated by dividing each participant’s fat mass (kg) by square of height (m). The cutoff value for high FMI was defined as 7.0 kg/m^2^, in accordance with previous studies [11, 12]. Central obesity was defined using waist circumference criteria from the Korean Society for the Study of Obesity (≥ 90 cm for men) [13]. Obesity was defined as BMI ≥ 25 kg/m^2^, based on criteria for individuals of Asian-Pacific ethnicity [14].

### Cardiovascular disease definition and assessment

ASCVD risk was estimated using the 10-year ASCVD risk score from the 2013 American College of Cardiology (ACC)/American Heart Association (AHA) guidelines [15]. According to this guideline, ten-year risk was defined as the risk of developing a first ASCVD event, defined as nonfatal myocardial infarction or coronary heart disease (CHD) death, or fatal or nonfatal stroke, over a 10-year period among people free from ASCVD at the beginning of the period. High probability of ASCVD risk was defined as ACC/AHA ASCVD risk > 20%. Participants were diagnosed with hypertension when their systolic pressure was ≥ 140 mmHg and/or their diastolic pressure was ≥ 90 mmHg, or if they currently used antihypertensive medications. Participants were diagnosed with diabetes mellitus (DM) if they used insulin or oral hypoglycemic agents, or if they had fasting plasma glucose ≥ 126 mg/dL and/or glycated hemoglobin ≥ 6.5%. Hyper-low-density lipoprotein (LDL) cholesterolemia was characterized based on LDL cholesterol goals recommended in the 2004 ATP III guidelines or the current use of an anti-dyslipidemia drug [16]. Hypo-high-density lipoprotein (HDL) cholesterolemia was defined as HDL < 40 mg/dL for men.

### Statistical analyses

The characteristics of the study participants were analyzed according to sarcopenia status using Student’s *t*-test or the Mann–Whitney *U* test for continuous variables, and the chi-square test or Fisher’s exact test for categorical variables. The association between sarcopenia index and ASCVD risk was evaluated using the chi-square test after variables had been transformed into quartiles. To control for the effects of central obesity or high FMI, participants were stratified into two groups depending on the presence of either central obesity or high FMI. Then, t-tests were used to compare ASCVD risk between groups and chi-square tests were conducted to assess associations between sarcopenia and proportion of participants with high ASCVD risk using odds ratios (ORs) with 95% confidence intervals (CIs). Multiple linear regression analyses were used to assess the independent effect of sarcopenia on ASCVD risk score. Multiple logistic regression analyses were used to assess the association between sarcopenia and high probability of ASCVD risk (ACC/AHA ASCVD 10-year risk score > 20%). Both multiple regression analyses were conducted via adjustment with other covariates (age, BMI, FEV_1_, current smoking, exercise, hypertension, DM, hyper-LDL cholesterolemia, hypo-HDL cholesterolemia, central obesity and FMI). Statistical analyses were performed using IBM SPSS Statistics for Windows, version 25.0 (IBM Corp., Armonk, NY, USA), and *P* < 0.05 was considered statistically significant.

## Results

### Baseline characteristics

In total, 704 participants were recruited (Fig. 1) and their baseline characteristics according to sarcopenia status are shown in Table 1. The prevalence of sarcopenia was 13.9% (98 of 704). Participants in the sarcopenia group were older, less likely to be current smokers, and had greater likelihoods of underlying diseases including hypertension, DM, and chronic kidney disease. Among the pulmonary function parameters, FVC, FVC% predicted, and FEV_1_ were lower in the sarcopenia group. Moreover, participants in the sarcopenia group had lower ASM, greater BMI, greater waist circumference, and greater proportions of central obesity and overall obesity. ASCVD risk score (27.5% vs. 16.3%, P < 0.001) and the proportion of participants with high ASCVD risk (69.5% vs. 30.9%, *P* < 0.001) were significantly greater in the sarcopenia group. Additional file 5: Table S1 shows the comparison between participants with low ASCVD risk and those with high ASCVD risk. The prevalence of high ASCVD risk was 36.2%. Among them, the prevalence of high ASCVD risk was 40.5% in the mild group, and 33.1% in the moderate to very severe group.

**Table 1:** Baseline characteristics of the study population

Variables	Non-sarcopenia	Sarcopenia*	*P*-value
	(n = 606)	(n = 98)	
Demographic variables			
Age, years	62.4 ± 10.9	69.7 ± 8.3	< 0.001
Height, cm	168.1 ± 5.6	161.2 ± 5.2	< 0.001
Weight, kg	65.9 ± 9.5	64.6 ± 10.3	0.210
BMI, kg/m^2^	23.2 ± 2.7	24.8 ± 3.0	< 0.001
Smoking status			
Never	63 (10.4)	8 (8.2)	0.011
Former	272 (44.9)	59 (60.2)	
Current	271 (44.7)	31 (31.6)	
Systolic BP, mmHg	123.3 ± 16.4	130.4 ± 16.2	< 0.001
Diastolic BP, mmHg	76.1 ± 10.9	74.2 ± 8.7	0.055
Hypertension	281 (46.4)	69 (70.4)	< 0.001
Diabetes mellitus	97 (16.4)	27 (28.7)	0.004
CKD	25 (4.1)	16 (16.3)	< 0.001
Regular exercise^a^	225 (37.3)	33 (33.7)	0.496
Body composition			
ASM, kg	21.0 ± 2.9	17.9 ± 2.2	< 0.001
Sarcopenic index (ASM/BMI)	0.9 ± 0.1	0.7 ± 0.04	< 0.001
Fat mass index, kg/m^2b^	5.0 ± 1.5	6.8 ± 1.7	< 0.001
High fat mass index^c^	54 (8.9)	37 (37.8)	< 0.001
Waist circumference, cm	84.7 ± 8.0	89.5 ± 8.8	< 0.001
Central obesity^d^	159 (26.3)	44 (44.9)	< 0.001
Obesity^e^	150 (24.8)	40 (40.8)	0.001
Laboratory variables			
Fasting blood glucose, mg/dL	103.7 ± 26.5	105.4 ± 19.8	0.526
Insulin, µIU/mL	9.1 ± 4.4	10.0 ± 4.2	0.054
HOMA-IR	2.4 ± 1.5	2.7 ± 1.3	0.069
Total cholesterol, mg/dL	184.6 ± 35.6	182.3 ± 37.6	0.561
Triglyceride, mg/dL	160.9 ± 165.1	165.5 ± 112.8	0.792
HDL cholesterol, mg/dL	45.6 ± 12.0	46.0 ± 11.8	0.051
LDL cholesterol, mg/dL	111.1 ± 34.3	100.6 ± 25.6	0.203
Serum creatinine, mg/dL	0.9 ± 0.2	1.0 ± 0.2	0.004
eGFR, mL/min/1.73 m^2^	89.3 ± 17.8	81.1 ± 17.8	< 0.001
Spirometry			
FVC, L	4.0 ± 0.8	3.3 ± 0.6	< 0.001
FVC, %	90.9 ± 14.0	84.1 ± 13.5	< 0.001
FEV_1_, L	2.5 ± 0.6	2.0 ± 0.5	< 0.001
FEV_1_, %	76.6 ± 15.9	75.8 ± 17.1	0.617
FEV_1_/FVC, %	62.1 ± 8.0	61.6 ± 8.2	0.632
FEV_1_ < 80% of predicted value	347 (57.3)	58 (59.2)	0.742
ASCVD			
ASCVD risk score, %	16.3 ± 11.4	27.5 ± 14.3	< 0.001
High ASCVD risk^f^	187 (30.9)	68 (69.5)	< 0.001

Variables are expressed as means ± standard deviations or n (%). BMI, body mass index; ASM, appendicular skeletal muscle; BP, blood pressure; CKD, chronic kidney disease; HOMA-IR, homeostatic model assessment of insulin resistance; HDL, high-density lipoprotein; LDL, low-density lipoprotein; eGFR, estimated glomerular filtration rate; PTH, parathyroid hormone; ALP, alkaline phosphate; FVC, forced vital capacity; FEV1, forced expiratory volume in 1 s; ASCVD, atherosclerotic cardiovascular disease

*Sarcopenia was defined as sarcopenia index < 0.774

^a^Regular exercise was defined as > 20 min per session and at least 3 times per week

^b^Fat mass index was calculated by dividing each participant’s fat mass (kg) by square of height (m) (kg/m^2^)

^c^High fat mass index was defined as fat mass index ≥ 7.0 kg/m^2^

^d^Central obesity was defined as waist circumference ≥ 90 cm

^e^Obesity was defined as BMI ≥ 25 kg/m^2^

^f^High ASCVD risk was defined as ASCVD score > 20%

### Association between sarcopenia index and ASCVD risk

**Fig. 2:**
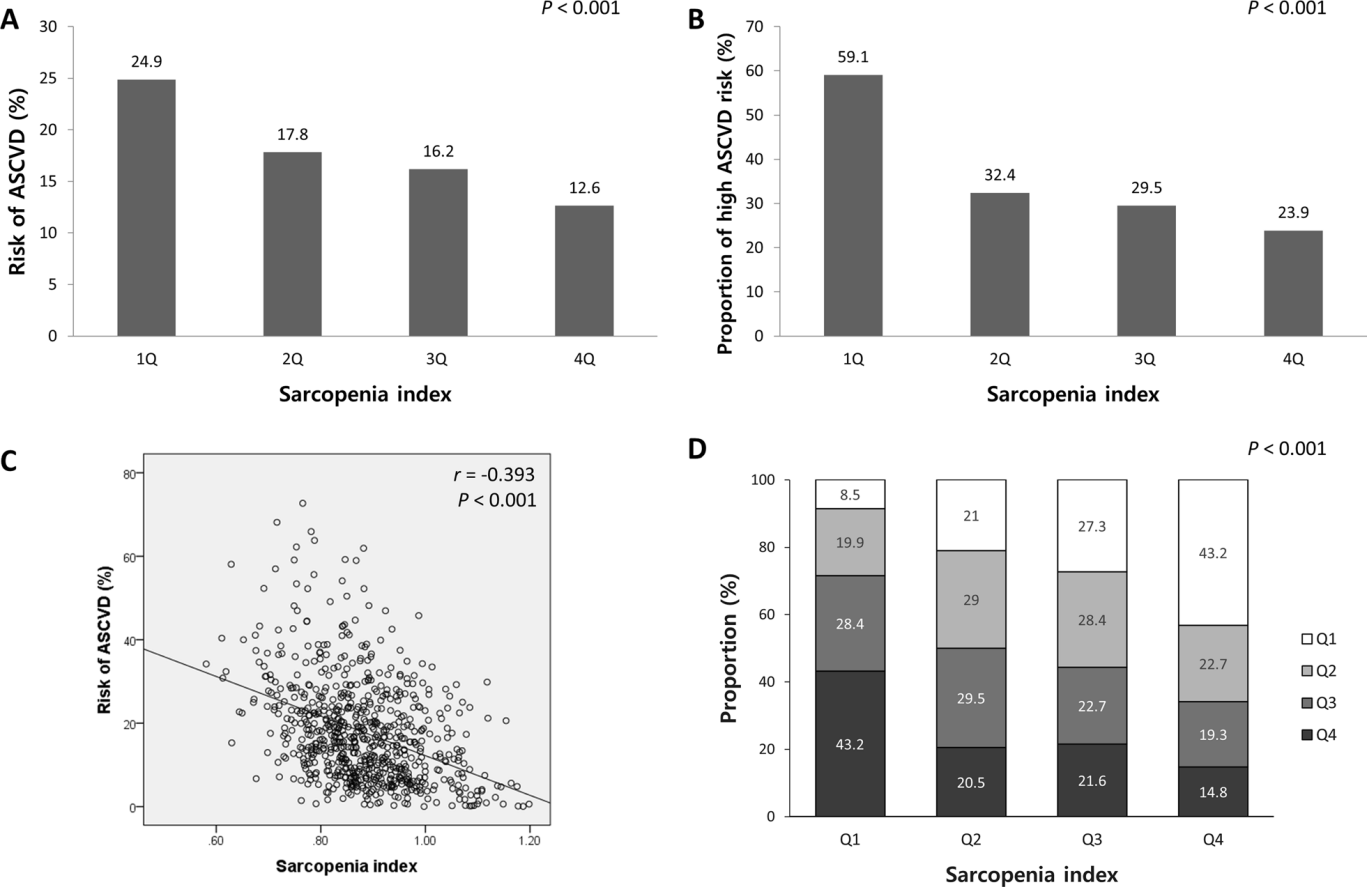
Association between sarcopenia index and ASCVD risk in quartile stratification analyses. The mean ASCVD risk (**A**) and proportion of participants with high ASCVD risk (**B**) both increased as the extent of sarcopenia increased (*P* < 0.001). **C** The sarcopenia index was negatively correlated with ASCVD risk (*P* < 0.001). **D** The sarcopenia index was negatively associated with ASCVD risk quartiles (*P* < 0.001). *ASCVD* atherosclerotic cardiovascular disease

We evaluated the association between sarcopenia index and ASCVD risk by quartile stratification of the sarcopenia index (Fig. 2). The mean ASCVD risk and proportion of participants with high ASCVD risk both increased with increasing extent of sarcopenia (Fig. 2A and B). There was a significant negative correlation between sarcopenia index and ASCVD risk (r = − 0.393, P < 0.001) (Fig. 2C). When ASCVD risk was stratified by quartiles, the sarcopenia index showed a strong negative relationship with ASCVD risk (P < 0.001) (Fig. 2D). The results of subgroup analyses according to airflow limitation severity are shown in Additional file 1: Fig. S1 (group with mild airflow limitation) and Additional file 1: Fig. S2 (group with moderate to very severe airflow limitation). Both groups with mild and moderate to very severe airflow limitations also showed similar negative associations between sarcopenia index and ASCVD risk.

### ASCVD risk and sarcopenic status stratified by central obesity and FMI

**Fig. 3:**
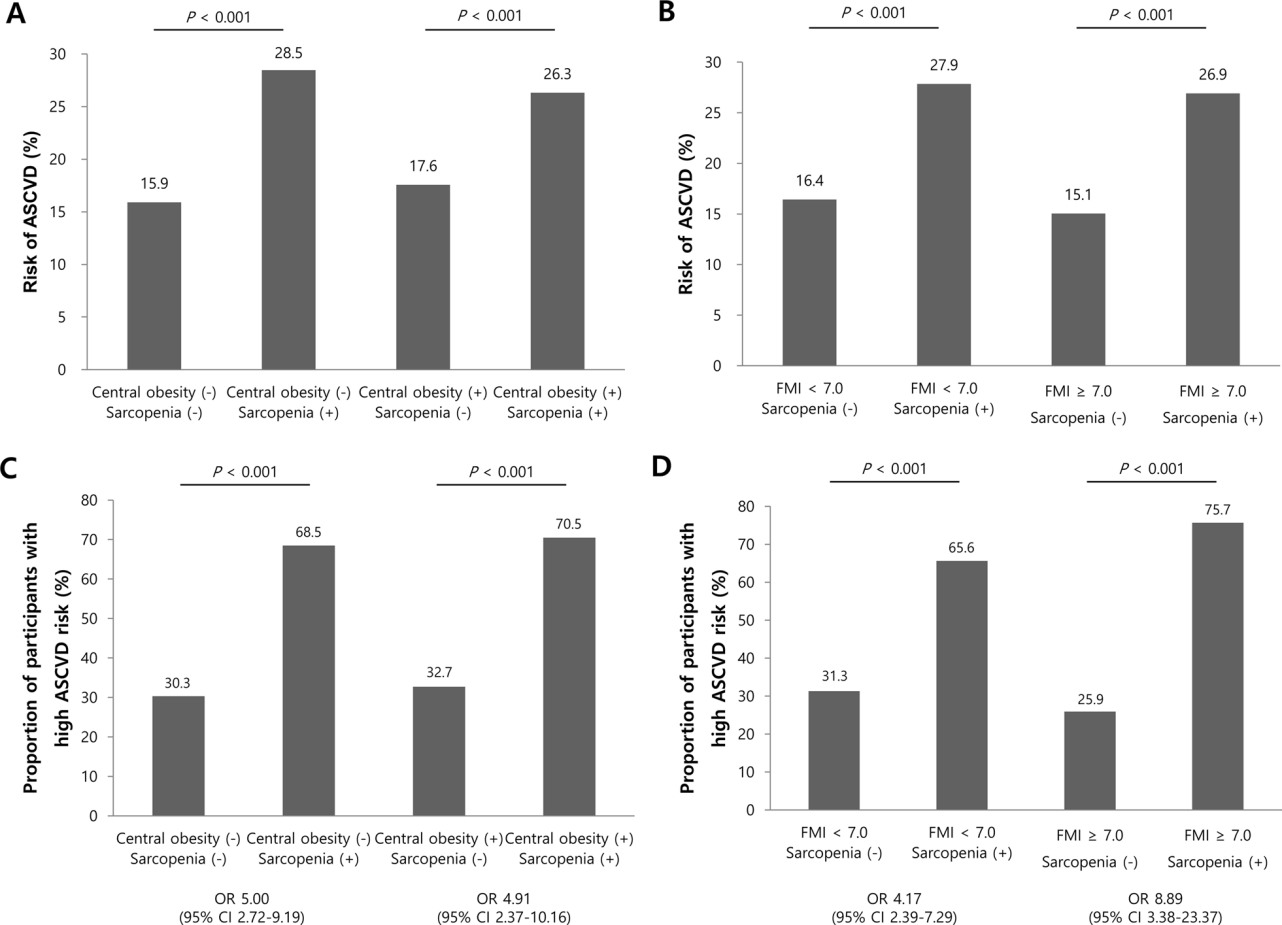
ASCVD risk and sarcopenic status stratified by central obesity and FMI. Associations between ASCVD risk and sarcopenia were assessed according to sarcopenic status, stratified by central obesity (**A**) and FMI (**B**). The prevalence of high ASCVD risk was evaluated according to sarcopenic status, stratified by central obesity (**C**) and FMI (**D**). ASCVD risk and proportion of participants with high ASCVD risk were greater in sarcopenic participants than in non-sarcopenic participants, regardless of central obesity and FMI (all *P* < 0.001). *ASCVD* atherosclerotic cardiovascular disease, *FMI* fat mass index, *OR* odds ratio, *CI* confidence interval

We evaluated ASCVD risk according to the presence of sarcopenia, without the confounding influences of obesity (as defined by central obesity and FMI) (Fig. 3). We stratified the study population using a cutoff value for waist circumference of 90 cm as central obesity (n = 499 [71.1%] with central obesity and 203 [28.9%] without central obesity) and a cutoff value for FMI of 7.0 kg/m^2^ (n = 613 [87.1%] with FMI < 7.0 kg/m^2^ and 91 [12.9%] with FMI ≥ 7.0 kg/m^2^) (Fig. 3). When the degree of cardiovascular risk was assessed using ASCVD risk score, the ASCVD risk score was greater among sarcopenic participants than among nonsarcopenic participants, regardless of central obesity (28.5% vs. 15.9% in participants without central obesity, 26.3% vs. 17.6% in participants with central obesity) (Fig. 3A) or FMI (mean 27.9% vs. 16.4% in participants with FMI < 7.0 kg/m^2^, mean 26.9% vs. 15.1% in participants with FMI ≥ 7.0 kg/m^2^) (Fig. 3B) (all *P* < 0.001).

We assessed the relative risk for high probability of ASCVD (ASCVD > 20%) according to the presence of sarcopenia in participants with and without central obesity or high FMI. A significantly greater proportion of sarcopenic participants had high ASCVD risk, compared to non-sarcopenic participants, regardless of central obesity (68.5% vs. 30.3% [OR = 5.00] in participants without central obesity; 70.5% vs. 32.7% [OR = 4.91] in participants with central obesity) (Fig. 3C) or FMI (65.6% vs. 31.3% [OR = 4.17] in participants with FMI < 7.0 kg/m^2^; 75.7% vs. 25.9% [OR = 8.89] in participants with FMI ≥ 7.0 kg/m^2^) (all *P* < 0.001) (Fig. 3D).

In subgroups with mild airflow limitation, sarcopenic participants had a higher ASCVD risk score only in those without central obesity or in those with FMI < 7.0 (Additional file 3: Fig. S3A and B). Moreover, there was a greater proportion of participants with high ASCVD risk only in those without central obesity (Additional file 3: Fig. S3C). However, sarcopenic participants had a greater proportion of participants with high ASCVD risk regardless of FMI (Additional file 3: Fig. S3D). In subgroups with moderate to very severe airflow limitation, sarcopenic participants had a higher ASCVD risk score and a greater proportion of participants with high ASCVD risk regardless of central obesity and FMI (all *P* < 0.001) (Additional file 4: Fig. S4).

### Independent association between sarcopenia and ASCVD risk

Multiple linear regression analyses were conducted to assess the independent association between sarcopenia and ASCVD risk (Table 2). Among the other risk factors, age, current smoking, hypertension, DM, and hypo-HDL cholesterolemia were significantly associated with ASCVD risk score (Table 2). Sarcopenia significantly increased ASCVD risk after adjustment for age, BMI, FEV_1_, current smoking, exercise, hypertension, DM, hyper-LDL cholesterolemia, hypo-HDL cholesterolemia, central obesity and FMI (estimates = 3.63; *P* < 0.001) (Table 2). In sub-groups stratified according to airflow limitation, sarcopenia was independently associated with ASCVD risk score in participants with mild and moderate to very severe airflow limitations (estimates = 2.63 in group with mild airflow limitation, *P* = 0.025; estimates = 4.29 in group with moderate to very severe airflow limitation, *P* < 0.001) (Table 2).

**Table 2:** Multiple linear regression analyses of ASCVD risk

Variables	All			Mild airflow limitation^d^			Moderate to very severe airflow limitation^e^		
	Estimated	SE	*P*-value	Estimated	SE	*P*-value	Estimated	SE	*P*-value
Age, years	0.78	0.03	< 0.001	0.93	0.04	< 0.001	0.71	0.03	< 0.001
BMI, kg/m^2^	− 0.01	− 0.23	0.816	0.01	0.25	0.805	− 0.01	− 0.46	0.647
Fat mass index, kg/m^2*^	0.01	0.08	0.939	0.01	0.27	0.790	− 0.01	− 0.37	0.710
Central obesity^a^	− 0.01	− 0.43	0.670	0.03	1.08	0.281	− 0.04	− 1.43	0.155
Current smoking	3.19	0.54	< 0.001	4.40	0.80	< 0.001	2.61	0.70	< 0.001
Regular exercise^b^	− 0.03	− 1.49	0.136	− 0.02	− 0.49	0.627	− 0.04	− 1.43	0.153
Hypertension	5.29	0.53	< 0.001	6.16	0.77	< 0.001	4.78	0.71	< 0.001
Diabetes mellitus	9.02	0.68	< 0.001	9.83	1.03	< 0.001	7.94	0.89	< 0.001
Hyper-LDL cholesterolemia	0.02	0.71	0.482	2.50	1.20	0.037	− 0.02	− 0.82	0.416
Hypo-HDL cholesterolemia	2.70	0.53	< 0.001	2.65	0.76	0.001	3.25	0.73	< 0.001
FEV_1_, L	0.01	0.15	0.885	0.01	− 0.01	0.627	0.03	0.83	0.405
Sarcopenia^c^	3.63	0.77	< 0.001	2.63	1.16	0.025	4.29	1.02	< 0.001

BMI, body mass index; FMI, fat mass index; LDL, low-density lipoprotein; HDL, high-density lipoprotein; FEV_1_, forced expiratory volume in 1 s; SE standard error

*Fat mass index was calculated by dividing each participant’s fat mass (kg) by square of height (m) (kg/m^2^)

^a^Central obesity was defined as waist circumference ≥ 90 cm in men

^b^Regular exercise was defined as > 20 min per session, at least 3 times per week (n = 702)

^c^Sarcopenia was defined as sarcopenia index < 0.774

^d^FEV_1_ ≥ 80% of predicted value

^e^FEV_1_ < 80% of predicted value

### Independent association between sarcopenia and high probability of ASCVD risk

The associations between high probability of ASCVD risk and presence of sarcopenia are shown in Table 3. Among the other risk factors, age, current smoking, hypertension, DM, hyper-cholesterolemia, and hypo-HDL cholesterolemia were independently associated with high probability of ASCVD risk (Table 3). Sarcopenia was significantly associated with high ASCVD risk after adjustments for other covariates (OR = 2.32, 95% CI 1.05–5.15; *P* = 0.039) (Table 3). In sub-groups stratified according to airflow limitation, sarcopenia was associated with high probability of ASCVD risk only in participants with moderate to very severe airflow limitation (OR = 2.97, 95% CI 1.05–5.15; *P* = 0.039) (Table 3).

**Table 3:** Multivariable logistic regression analyses to predict high probability of ASCVD risk

Variables	All			Mild airflow limitation^d^			Moderate to very severe airflow limitation^e^		
	OR	95% CI	*P*-value	OR	95% CI	*P*-value	OR	95% CI	*P*-value
Age, years	1.56	1.44–1.681	< 0.001	1.55	1.38–1.73	< 0.001	1.59	1.42–1.77	< 0.001
BMI, kg/m^2^	1.11	0.91–1.350	0.295	1.21	0.88–1.64	0.21	1.06	0.81–1.39	0.683
Fat mass index, kg/m^2*^	0.93	0.67–1.303	0.686	0.91	0.57–1.47	0.704	0.95	0.58–1.57	0.846
Central obesity^a^	0.84	0.38–1.884	0.677	0.86	0.25–3.00	0.818	0.78	0.24–2.37	0.655
Current smoking	3.74	2.06–6.779	< 0.001	6.85	2.43–19.31	< 0.001	3.06	1.33–6.71	0.005
Regular exercise^b^	1.01	0.57–1.787	0.969	0.76	0.33–1.76	0.516	1.26	0.56–2.85	0.582
Hypertension	8.42	4.52–15.69	< 0.001	11.64	4.58–29.60	< 0.001	8.98	3.73–21.63	< 0.001
Diabetes mellitus	13.77	6.40–29.50	< 0.001	18.06	5.72–57.06	< 0.001	16.06	5.38–47.92	< 0.001
Hyper-LDL cholesterolemia	3.11	1.22–7.92	0.018	5.60	1.42–22.02	0.014	2.20	0.60–8.07	0.237
Hypo-HDL cholesterolemia	3.03	1.71–5.36	< 0.001	3.48	1.50–8.08	0.004	2.77	1.24–6.22	0.013
FEV_1_, L	1.38	0.82–2.33	0.231	1.33	0.37–4.77	0.664	1.04	0.40–2.70	0.937
Sarcopenia^c^	2.32	1.05–5.15	0.039	1.93	0.41–9.15	0.41	2.97	1.06–8.36	0.039

*ASCVD* atherosclerotic cardiovascular disease, *BMI* body mass index, *LDL* low-density lipoprotein, *HDL* high-density lipoprotein, *FEV*_*1*_ forced expiratory volume in 1 s, *OR* odds ratio, *CI* confidence interval

*Fat mass index was calculated by dividing each participant’s fat mass (kg) by square of height (m) (kg/m^2^)

^a^Central obesity was defined as waist circumference ≥ 90 cm in men

^b^Regular exercise was defined as > 20 min per session, at least 3 times per week (n = 702)

^c^Sarcopenia was defined as sarcopenia index < 0.774

^d^FEV_1_ ≥ 80% of predicted value

^e^FEV_1_ < 80% of predicted value

## Discussion

High ASCVD risk became more prevalent as the extent of sarcopenia increased in COPD patients. Sarcopenic men with COPD, particularly those with moderate to very severe airflow limitation, had an approximately three-fold greater 10-year risk for ASCVD events, according to ASCVD risk equations. This risk was independent of fat mass, central obesity, hyper-LDL cholesterolemia, DM, hypertension, amount of exercise, and current smoking. However, this significant risk was not observed in patients with COPD who had mild airflow limitation. To the best of our knowledge, no other study has assessed the impact of coexisting sarcopenia, with or without high fat mass or central obesity, on ASCVD risk in patients with COPD. These results suggest that sarcopenia is closely associated with an increased risk for ASCVD, regardless of high fat mass or central obesity, in patients with COPD who have moderate to very severe airflow limitation.

Sarcopenia is common in patients with COPD (prevalence of 7.9–66.7%) [17]. According to the previous study based on the data of Korean NHANES, sarcopenia was more prevalent in patients with COPD than in the general population [3]. Muscle loss is associated with physical inactivity or disuse, hypoxemia, malnutrition, and systemic inflammation [3]. Sarcopenia in patients with COPD is associated with reduced lung function, poor quality of life, poor exercise tolerance, and low bone marrow density [3, 18]. Moreover, the prevalence of sarcopenia increases with increasing severity of airflow limitation [3]. In relation to ASCVD risk, rehabilitation-induced changes in cardiometabolic risk markers such as LDL cholesterol and homeostasis model assessment of insulin resistance (HOMA-IR) are reportedly sarcopenia-dependent [2]. Increased LDL cholesterol level and HOMA-IR score are related to increased rates of cardiovascular events and mortality [12]. In study based on the data of KNHANES, Sarcopenia was associated with the presence of CVD independent of other cardiovascular risk factors in the subjects older than 65 years of age, and the odds ratio was 1.77 [19]. However, the impact of sarcopenia on ASCVD risk in patients with COPD has not been fully evaluated.

The relationship between sarcopenia and increased ASCVD risk may involve several mechanisms. Skeletal muscle is the primary systemic tissue responsible for glucose uptake and deposition, as well as myokine secretion, which plays a protective role against insulin resistance, independent of obesity [20]. The association between sarcopenia with insulin resistance and DM might be an intermediate step in the development of frailty in individuals with sarcopenia [20, 21]. Moreover, individuals with lower muscle mass have increased risks of nonalcoholic fatty liver disease and liver fibrosis, which are features of metabolic syndrome [5, 14]. In addition to the relationship between sarcopenia and risk for metabolic syndrome, sarcopenia is associated with cardiovascular risk factors such as hypertension and arterial stiffness [22, 23]. The coexistence of sarcopenia and metabolic syndrome is reportedly associated with increased risks of type 2 DM, hypertension arterial stiffness, and hyperlipidemia in Japanese women [24]. Moreover, circulating markers of oxidative stress are increased in the context of sarcopenia and related to cardiovascular disease risk in patients with sarcopenic obesity [25]. However, no studies have evaluated the effects of sarcopenia on oxidative stress, despite convincing published evidence regarding pathophysiological changes in patients with COPD.

The impact of sarcopenia on increased risk for ASCVD was statistically significant in men with COPD who had moderate to very severe airflow limitation, but not in patients who had mild airflow limitation. The sarcopenia index did not differ between groups and was not associated with airflow limitation (data not shown), consistent with previous findings [26, 27]. However, a loss of muscle mass can result in decreased muscle oxidative capacity, which may be more pronounced in patients with COPD due to a muscle fiber I to II shift [28, 29]. Moreover, COPD is accompanied by systemic inflammation and oxidative stress, which increase with greater severity of airflow limitation [30, 31]. Among sarcopenic patients with COPD who have moderate to severe airflow limitation, most showed lower physical function [28]. Muscle strength in sarcopenic patients reduces the risk for cardiovascular disease from 23 to 18% after controlling for physical activity. This suggests that the pathway through which sarcopenia affects cardiovascular disease risk is at least partly mediated by physical activity [32]. Therefore, the effects of sarcopenia on the risk for ASCVD are presumably more prominent in patients with COPD who have low physical activity due to severe airflow limitation.

In contrast to sarcopenia, FMI and central obesity were not associated with ASCVD risk in this study. Obesity is an important health problem that increases the risks of cardiovascular and metabolic diseases. Participants with sarcopenic obesity exhibited a greater risk for ASCVD, compared to participants who had a normal BMI and muscle mass, but participants with non-sarcopenic obesity or non-obese sarcopenia did not exhibit a greater risk for ASCVD in a study of the general population [6]. However, obesity-based BMI can incorrectly categorize individuals as obese, particularly those who have high muscle mass without excess body fat. Moreover, central adipose tissue was not associated with the incidence of ASCVD events in older individuals [33]. This is consistent with the “obesity paradox” hypothesis, whereby adipose tissue depots may be associated with greater vitality in advanced age, resulting in better survival and improved functional outcomes [28, 33]. Sarcopenic patients with COPD who had concomitant central obesity reportedly showed greater physical function, compared to sarcopenic patients with COPD who did not have central obesity [32]. In the present study, a greater prevalence of central obesity was observed in sarcopenic patients with COPD than in non-sarcopenic patients with COPD. The excess ASCVD risk caused by central obesity or high fat mass might become less important than the risk caused by other factors in older and sarcopenic patients with COPD.

Therefore, our study has clinical significance, because we conducted a comprehensive risk comparison using various clinical and metabolic factors to evaluate the association between sarcopenia and cardiovascular disease risk. These factors included current smoking, hypertension, DM, hyper-LDL cholesterolemia, central obesity, and FMI, which are known cardiovascular disease risk factors. Major age-related changes in body composition include an increase in body fat and a decline in skeletal muscle, although BMI may remain relatively constant [34]. Therefore, we included FMI and central obesity as covariates, although previous studies used BMI to assess obesity. The mean age of patients with COPD in our study was > 60 years, suggesting that BMI would not accurately assess obesity in this population. Moreover, data from a similar nationwide survey in the United States showed that normal-weight central obesity was associated with greater ASCVD mortality, compared to BMI-defined obesity [35].

However, the present study had several potential limitations. First, due to the cross-sectional nature of the data, causal relationships between sarcopenia and ASCVD risk could not be identified. Second, the 10-year ASCVD risk was estimated by the ASCVD risk equation, instead of by assessment of real ASCVD events. Although the ASCVD risk equations were well-calibrated and validated in a population of individuals in the United States, our findings should be interpreted cautiously because individuals with pure Asian ethnicity were not present in the cohort used for development of ASCVD risk equations [36, 37]. However, the findings by Chia et al. suggest better discrimination of ASCVD risk prediction in the general population because moderate discrimination and good calibration of ASCVD risk equation were observed in the primary care setting in an Asian population [38]. Third, we defined sarcopenia based on the ASM index alone, not on muscle function (e.g., muscle strength physical performance) [39]. Current recommendations for sarcopenia assessment include thorough evaluation of three domains of muscle mass, muscle strength, and physical performance. However, absolute cutoff levels to predict sarcopenia have not been established for these domains. Therefore, this study used the adjusted level of exercise for analyses. Fourth, other potential confounding factor, such as vitamin D status, was not available in this study, which is risk for both cardiovascular disease and sarcopenia. Fifth, the results cannot be generalized to women with COPD because only men were included in the present study. In women, bone density is greatly influenced by sex hormone levels, which decrease drastically after menopause. Because factors affecting muscle mass and bone density can differ according to sex, women participants were not included in our study. Sixth, the cut-off value of < 0.774 to define sarcopenia was lower than that of general elderly subjects because the subjects of this study were COPD patients who already had muscle loss. Lastly, because the number of subjects in the severe—very severe COPD group was too small, the statistical significance for this group compared to other groups could not be concluded in this study.

## Conclusions

In conclusion, sarcopenia was significantly associated with increased ASCVD risk in men with COPD, particularly those with moderate to very severe airflow limitation, independent of other clinical and metabolic factors (e.g., central obesity and FMI). Our study suggests that physicians should evaluate skeletal muscle status to identify men with COPD who have a high risk for cardiovascular disease. However, further studies are warranted to elucidate the causal relationship between sarcopenia and cardiovascular disease in men with COPD.

## Supplementary Information

**Additional file 1: Figure S1. **Association between sarcopenia index and ASCVD risk by quartile stratification analyses in participants with mild airflow limitation. The mean ASCVD risk (A) and proportion of participants with high ASCVD risk (B) both increased as the extent of sarcopenia increased (*P* < 0.001). (C) The sarcopenia index was negatively associated with ASCVD risk quartiles (*P* < 0.001). ASCVD, atherosclerotic cardiovascular disease

**Additional file 2: Figure S2. **Association between sarcopenia index and ASCVD risk by quartile stratification analyses in participants with moderate to very severe airflow limitation. The mean ASCVD risk (A) and proportion of participants with high ASCVD risk (B) both increased as the extent of sarcopenia increased (*P* < 0.001). (C) The sarcopenia index was negatively associated with ASCVD risk quartiles (*P* < 0.001). ASCVD, atherosclerotic cardiovascular disease

**Additional file 3: Figure S3. **ASCVD risk and sarcopenic status stratified by central obesity and FMI in participants with mild airflow limitation. Associations between ASCVD risk and sarcopenia were assessed according to sarcopenic status, stratified by central obesity (A) and FMI (B). Sarcopenic participants had a higher ASCVD risk score only in those without central obesity or in those with FMI < 7.0. The prevalence of high ASCVD risk was evaluated according to sarcopenic status, stratified by central obesity (C) and FMI (D). There was a greater proportion of participants with high ASCVD risk only in those without central obesity. However, sarcopenic participants had a greater proportion of participants with high ASCVD risk regardless of FMI. ASCVD, atherosclerotic cardiovascular disease; FMI, fat mass index; OR, odds ratio; CI, confidence interval

**Additional file 4: Figure S4. **ASCVD risk and sarcopenic status stratified by central obesity and FMI in participants with moderate to very severe airflow limitation. Associations between ASCVD risk and sarcopenia were assessed according to sarcopenic status, stratified by central obesity (A) and FMI (B). The prevalence of high ASCVD risk was evaluated according to sarcopenic status, stratified by central obesity (C) and FMI (D). ASCVD risk and proportion of participants with high ASCVD risk were greater in sarcopenic participants than in non-sarcopenic participants, regardless of central obesity and FMI (all *P* < 0.001). ASCVD, atherosclerotic cardiovascular disease; FMI, fat mass index; OR, odds ratio; CI, confidence interval.

**Additional file 5: Table S1.** Baseline characteristics of the study population

## Data Availability

The datasets used and/or analysed during the current study are available from the Korea National Health and Nutrition Examination Survey database on reasonable request (https://knhanes.kdca.go.kr/knhanes/eng/index.do).

## Abbreviations

ASCVD: Atherosclerotic cardiovascular disease
COPD: Chronic obstructive pulmonary disease
BMI: Body mass index
KNHANES: Korea National Health and Nutrition Examination Survey
FEV_1_: Forced expiratory volume in 1 s
FVC: Forced vital capacity
ASM: Appendicular skeletal muscle
FMI: Fat mass index
ACC: American College of Cardiology
AHA: American Heart Association
DM: Diabetes mellitus
LDL: Low-density lipoprotein
HDL: High-density lipoprotein
OR: Odds ratio
CI: Confidence interval
