# Supplementary figures and images for: Sarcopenia is associated with cardiovascular risk in men with COPD, independent of adiposity
Source: Respir Res. 2022 Jul 13;23:185. doi: 10.1186/s12931-022-02109-3 (PMC9281034; doi:10.1186/s12931-022-02109-3)

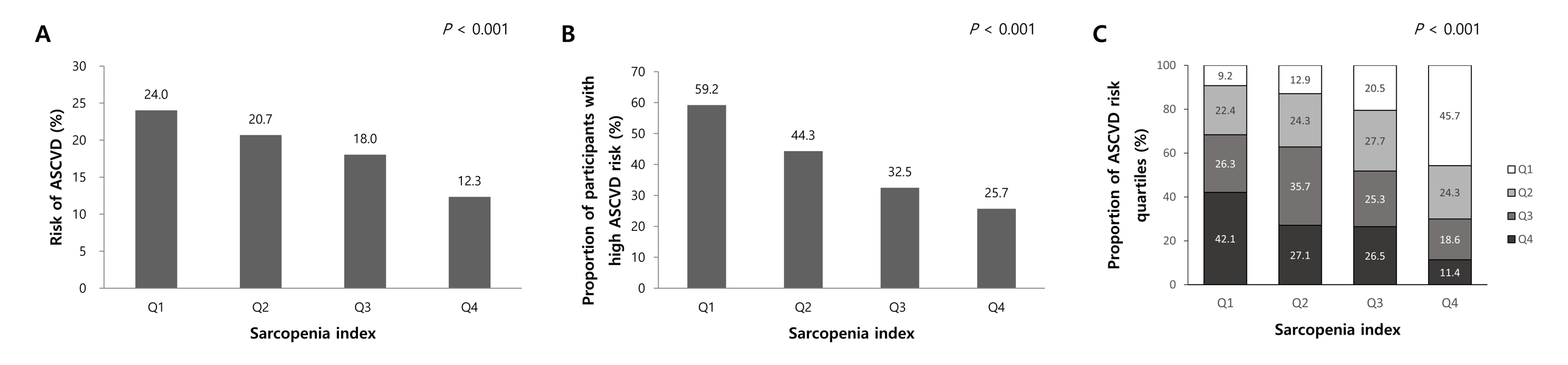

Supplement: Supplementary file 1 — Additional file 1: Figure S1. Association between sarcopenia index and ASCVD risk by quartile stratification analyses in participants with mild airflow limitation. The mean ASCVD risk (A) and proportion of participants with high ASCVD risk (B) both increased as the extent of sarcopenia increased (P < 0.001). (C) The sarcopenia index was negatively associated with ASCVD risk quartiles (P < 0.001). ASCVD, atherosclerotic cardiovascular disease [file 12931_2022_2109_MOESM1_ESM.tif]

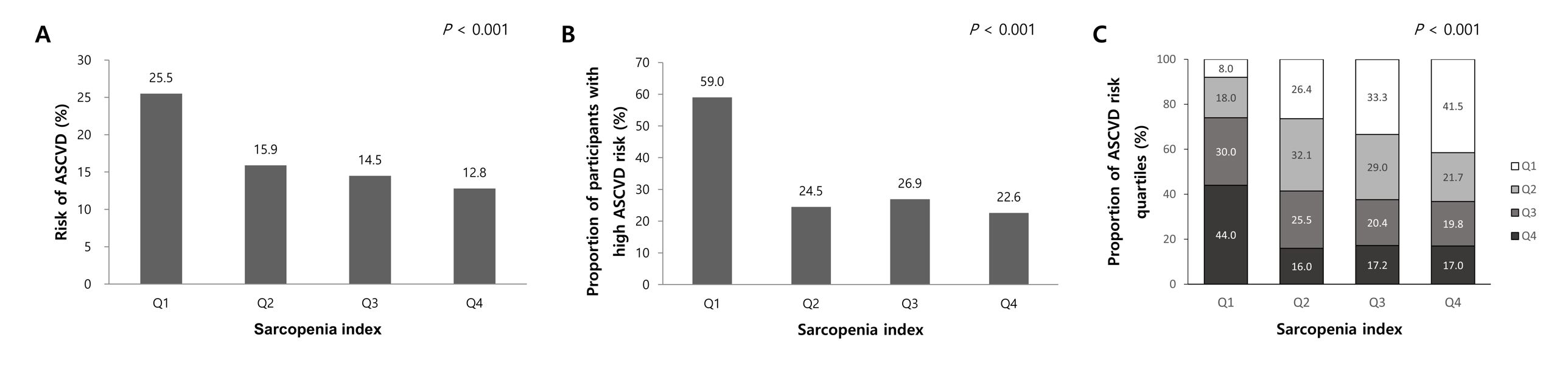

Supplement: Supplementary file 2 — Additional file 2: Figure S2. Association between sarcopenia index and ASCVD risk by quartile stratification analyses in participants with moderate to very severe airflow limitation. The mean ASCVD risk (A) and proportion of participants with high ASCVD risk (B) both increased as the extent of sarcopenia increased (P < 0.001). (C) The sarcopenia index was negatively associated with ASCVD risk quartiles (P < 0.001). ASCVD, atherosclerotic cardiovascular disease [file 12931_2022_2109_MOESM2_ESM.tif]

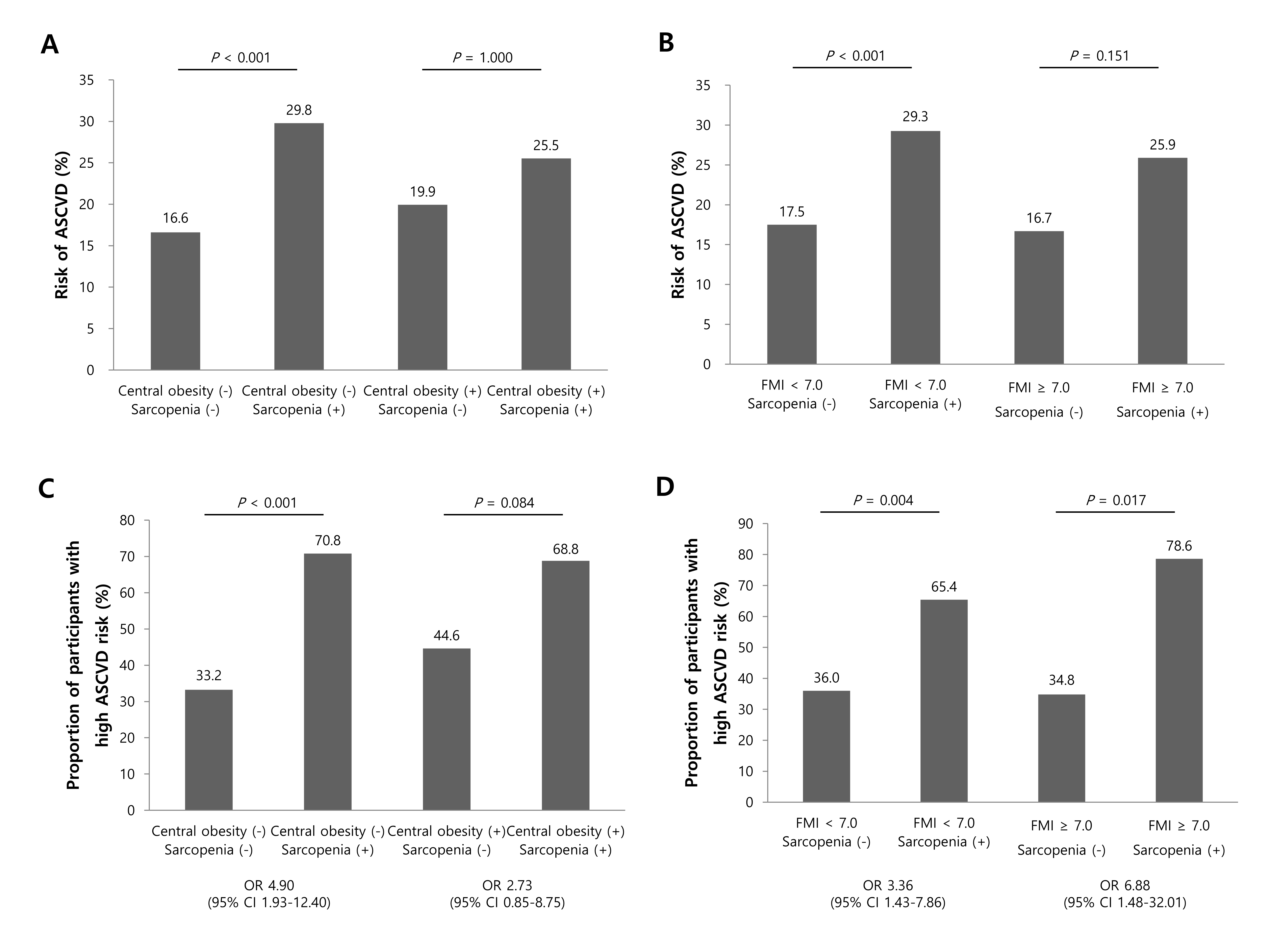

Supplement: Supplementary file 3 — Additional file 3: Figure S3. ASCVD risk and sarcopenic status stratified by central obesity and FMI in participants with mild airflow limitation. Associations between ASCVD risk and sarcopenia were assessed according to sarcopenic status, stratified by central obesity (A) and FMI (B). Sarcopenic participants had a higher ASCVD risk score only in those without central obesity or in those with FMI < 7.0. The prevalence of high ASCVD risk was evaluated according to sarcopenic status, stratified by central obesity (C) and FMI (D). There was a greater proportion of participants with high ASCVD risk only in those without central obesity. However, sarcopenic participants had a greater proportion of participants with high ASCVD risk regardless of FMI. ASCVD, atherosclerotic cardiovascular disease; FMI, fat mass index; OR, odds ratio; CI, confidence interval [file 12931_2022_2109_MOESM3_ESM.tif]

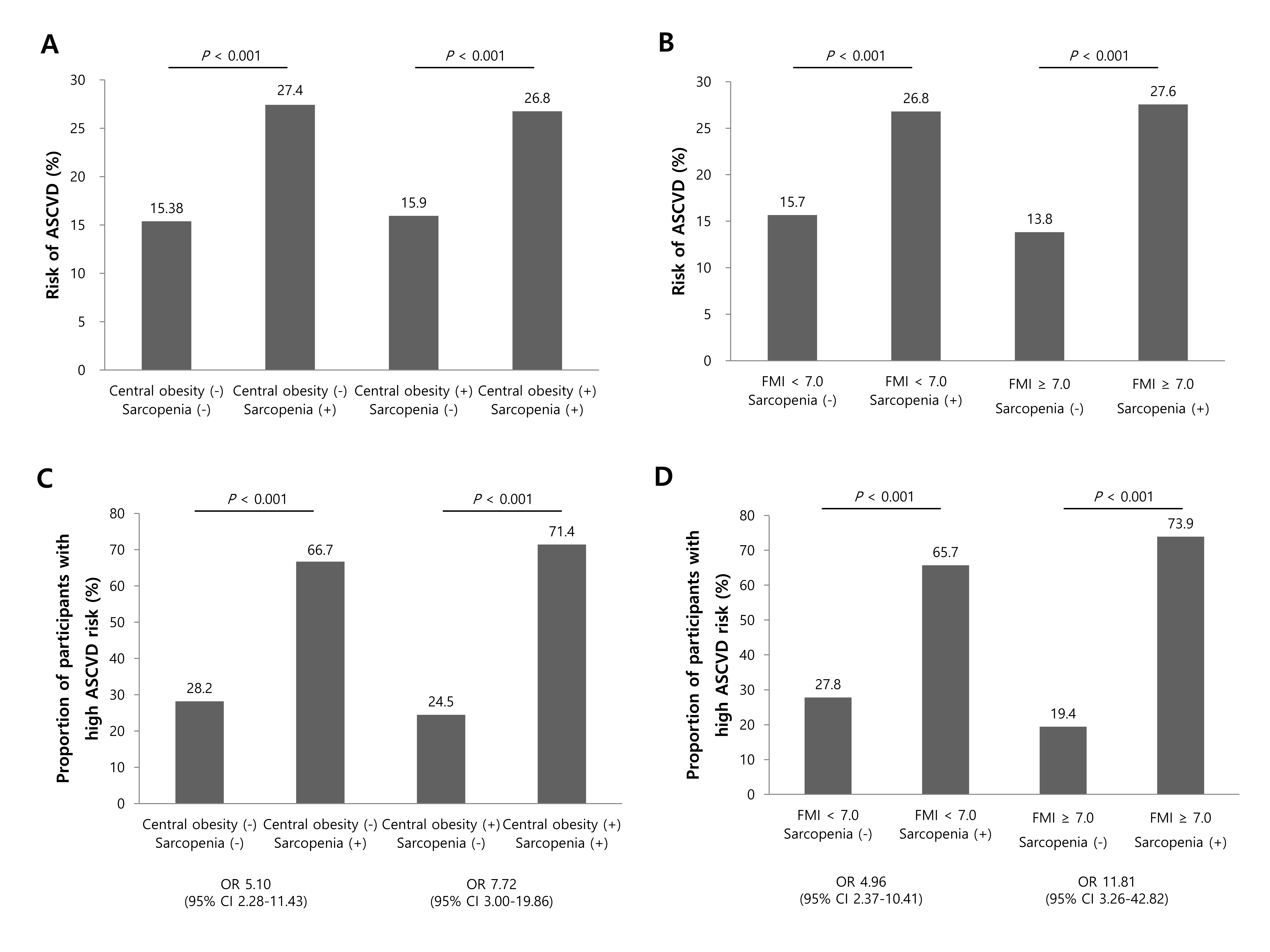

Supplement: Supplementary file 4 — Additional file 4: Figure S4. ASCVD risk and sarcopenic status stratified by central obesity and FMI in participants with moderate to very severe airflow limitation. Associations between ASCVD risk and sarcopenia were assessed according to sarcopenic status, stratified by central obesity (A) and FMI (B). The prevalence of high ASCVD risk was evaluated according to sarcopenic status, stratified by central obesity (C) and FMI (D). ASCVD risk and proportion of participants with high ASCVD risk were greater in sarcopenic participants than in non-sarcopenic participants, regardless of central obesity and FMI (all P < 0.001). ASCVD, atherosclerotic cardiovascular disease; FMI, fat mass index; OR, odds ratio; CI, confidence interval. [file 12931_2022_2109_MOESM4_ESM.tif]
