# Supplementary material for: Sarcopenia is associated with cardiovascular risk in men with COPD, independent of adiposity
Source: Respir Res. 2022 Jul 13;23:185. doi: 10.1186/s12931-022-02109-3 (PMC9281034; doi:10.1186/s12931-022-02109-3)
Supplement: Supplementary file 5 — Additional file 5: Table S1. Baseline characteristics of the study population [file 12931_2022_2109_MOESM5_ESM.docx]

**Table S1. Baseline characteristics of the study population**

| **Variables** | **Low ASCVD risk** | **High ASCVD risk^*^** | ***P*-value** |
| --- | --- | --- | --- |
|  | **(n = 449)** | **(n = 255)** |  |
| Demographic variables |  |  |  |
| Age, years | 58.5 ± 10.2 | 72.1 ± 5.5 | < 0.001 |
| Height, cm | 168.1 ± 5.9 | 165.4 ± 5.6 | < 0.001 |
| Weight, kg | 66.6 ± 10.0 | 64.2 ± 8.7 | 0.001 |
| BMI, kg/m^2^ | 23.5 ± 2.9 | 23.4 ± 2.6 | 0.677 |
| Smoking status, current smoker | 210 (46.9) | 99 (39.0) | 0.043 |
| Systolic BP, mmHg | 110.6 ± 15.4 | 132.5 ± 15.2 | < 0.001 |
| Diastolic BP, mmHg | 76.0 ± 11.1 | 75.6 ± 9.8 | 0.613 |
| Hypertension | 173 (38.5) | 177 (69.4) | < 0.001 |
| Diabetes mellitus | 45 (10.4) | 79 (31.5) | < 0.001 |
| CKD | 12 (2.7) | 29 (11.4) | < 0.001 |
| Regular exercise^†^ | 176 (39.3) | 82 (32.3) | 0.064 |
| Body composition |  |  |  |
| ASM, kg | 21.2 ± 3.0 | 19.6 ± 2.6 | < 0.001 |
| Sarcopenic index (ASM/BMI) | 0.9 ± 0.1 | 0.8 ± 0.1 | < 0.001 |
| Sarcopenia^‡^ | 30 (6.7) | 68 (26.7) | < 0.001 |
| Fat mass index, kg/m^2§^ | 5.1 ± 1.6 | 5.5 ± 1.7 | 0.003 |
| High fat mass index^∥^ | 49 (10.9) | 42 (16.5) | 0.035 |
| Waist circumference, cm | 84.9 ± 8.3 | 86.3 ± 8.2 | 0.026 |
| Central obesity^**^ | 120 (26.8) | 83 (32.5) | 0.109 |
| Obesity^††^ | 132 (29.4) | 58 (22.7) | 0.056 |
| Laboratory variables |  |  |  |
| Fasting blood glucose, mg/dL | 101.0 ± 21.9 | 109.1 ± 30.7 | < 0.001 |
| Insulin, µIU/mL | 9.1 ± 4.3 | 9.3 ± 4.5 | 0.514 |
| HOMA-IR | 2.3 ± 1.6 | 2.5 ± 1.4 | 0.087 |
| Total cholesterol, mg/dL | 183.6 ± 34.1 | 185.5 ± 38.9 | 0.490 |
| Triglyceride, mg/dL | 154.2 ± 114.3 | 174.6 ± 215.5 | 0.102 |
| HDL cholesterol, mg/dL | 50.4 ± 12.3 | 44.4 ± 10.4 | < 0.001 |
| LDL cholesterol, mg/dL | 107.4 ± 33.2 | 116.2 ± 33.8 | 0.136 |
| Serum creatinine, mg/dL | 0.9 ± 0.2 | 1.0 ± 0.2 | < 0.001 |
| eGFR, mL/min/1.73 m^2^ | 92.1 ± 17.2 | 81.4 ± 17.3 | < 0.001 |
| Spirometry |  |  |  |
| FVC, L | 4.1 ± 0.7 | 3.6 ± 0.7 | < 0.001 |
| FVC, % | 92.1 ± 13.4 | 86.2 ± 14.6 | < 0.001 |
| FEV_1_, L | 2.6 ± 0.6 | 2.2 ± 0.6 | < 0.001 |
| FEV_1_, % | 76.1 ± 15.2 | 77.3 ± 17.5 | 0.321 |
| FEV_1_/FVC, % | 62.4 ± 7.6 | 61.3 ± 8.7 | 0.080 |
| FEV_1_ < 80% of predicted value | 271 (60.4) | 134 (52.5) | 0.044 |
| ASCVD |  |  |  |
| ASCVD risk score, % | 10.4 ± 5.5 | 31.0 ± 10.3 | < 0.001 |

Variables are expressed as means ± standard deviations or n (%). BMI, body mass index; ASM, appendicular skeletal muscle; BP, blood pressure; CKD, chronic kidney disease; HOMA-IR, homeostatic model assessment of insulin resistance; HDL, high-density lipoprotein; LDL, low-density lipoprotein; eGFR, estimated glomerular filtration rate; PTH, parathyroid hormone; ALP, alkaline phosphate; FVC, forced vital capacity; FEV1, forced expiratory volume in 1 s; ASCVD, atherosclerotic cardiovascular disease.

**^*^**High ASCVD risk was defined as ASCVD score > 20%

^†^Regular exercise was defined as >20 min per session and at least 3 times per week

^‡^Sarcopenia was defined according to the presence of sarcopenia index < 0.774

^§^Fat mass index was calculated by dividing each participant’s fat mass (kg) by square of height (m) (kg/m^2^)

^∥^High fat mass index was defined as fat mass index ≥ 7.0 kg/m^2^

^**^Central obesity was defined as waist circumference ≥ 90 cm; ^††^Obesity was defined as BMI ≥ 25 kg/m
